# The impact of partnered pharmacist medication charting in the emergency department on the use of potentially inappropriate medications in older people

**DOI:** 10.3389/fphar.2023.1273655

**Published:** 2023-11-07

**Authors:** Tesfay Mehari Atey, Gregory M. Peterson, Mohammed S. Salahudeen, Barbara C. Wimmer

**Affiliations:** School of Pharmacy and Pharmacology, College of Health and Medicine, University of Tasmania, Hobart, Australia

**Keywords:** partnered pharmacist, co-charting, medication charting, emergency department, potentially inappropriate medication

## Abstract

**Introduction:** A process redesign, partnered pharmacist medication charting (PPMC), was recently piloted in the emergency department (ED) of a tertiary hospital. The PPMC model was intended to improve medication safety and interdisciplinary collaboration by having pharmacists work closely with medical officers to review and chart medications for patients. This study, therefore, aimed to evaluate the impact of PPMC on potentially inappropriate medication (PIM) use.

**Methods:** A pragmatic concurrent controlled study compared a PPMC group to both early best-possible medication history (BPMH) and usual care groups. In the PPMC group, pharmacists initially documented the BPMH and collaborated with medical officers to co-develop treatment plans and chart medications in ED. The early BPMH group included early BPMH documentation by pharmacists, followed by traditional medication charting by medical officers in ED. The usual care group followed the traditional charting approach by medical officers, without a pharmacist-collected BPMH or collaborative discussion in ED. Included were older people (≥65 years) presenting to the ED with at least one regular medication with subsequent admission to an acute medical unit. PIM outcomes (use of at least one PIM, PIMs per patient and PIMs per medication prescribed) were assessed at ED presentation, ED departure and hospital discharge using Beers criteria.

**Results:** Use of at least one PIM on ED departure was significantly lower for the PPMC group than for the comparison groups (χ^2^, *p* = 0.040). However, PIM outcomes at hospital discharge were not statistically different between groups. PIM outcomes on ED departure or hospital discharge did not differ from baseline within the comparison groups.

**Discussion:** In conclusion, PIM use on leaving ED, but not at hospital discharge, was reduced with PPMC. Close interprofessional collaboration, as in ED, needs to continue on the wards.

## 1 Introduction

Improving patient care requires a focus on medication safety in the emergency department (ED), a setting characterized by heavy workloads and complex medication use processes (Brown, 2005; Juarez et al., 2009). Evidence shows that up to 32% of older people presenting to the ED may have at least one potentially inappropriate medication (PIM) in their home medicines (Hustey et al., 2007). The term “PIM”, operationalized using evidence-based criteria and expert opinions (Hanlon and Schmader, 2013; O'Mahony et al., 2015; American Geriatrics Society Beers Criteria, 2019), suggests that the risk of a medication potentially outweighs its benefits, and the medication should generally be avoided. Exposure to PIMs can increase the risk of adverse drug events, ED revisits and rehospitalization, leading to increased healthcare costs (Dalleur et al., 2012; Dormann et al., 2013; Weir et al., 2020; Schiavo et al., 2022).

The inclusion of pharmacists in prescribing models has gained popularity over the past quarter-century globally, with emphasis on strategies to improve medication safety (Galt, 1995; Gray, 2002; Kay and Brien, 2004; Tonna et al., 2007; Stewart et al., 2008). Involving pharmacists in ED care through collaborative charting models is one of the strategies advocated to potentially improve medication safety (Vasileff et al., 2009; Taylor et al., 2019; Atey et al., 2022; Atey et al., 2023). An example is partnered pharmacist medication charting (PPMC), which refers to the co-charting of a patient’s medicines by a pharmacist following a clinical conversation with a medical officer who is in charge of the patient care (Tong et al., 2016). While previous studies in Australia have primarily focused on the impact of PPMC on medication errors and length of hospital stay (Vasileff et al., 2009; Khalil et al., 2016; Tong et al., 2020; Atey et al., 2023), there remains a notable gap in the national and global literature on whether PPMC has any impact on the use of PIMs. Therefore, this pragmatic controlled study investigated the impact of PPMC on the use of PIMs in older people.

## 2 Materials and methods

The study’s methodology (Sections 2.1–2.6) including the study setting, period, design, groups, inclusion/exclusion criteria and data collection procedures, are published elsewhere in detail (Atey et al., 2023).

### 2.1 PPMC

**FIGURE 1:**
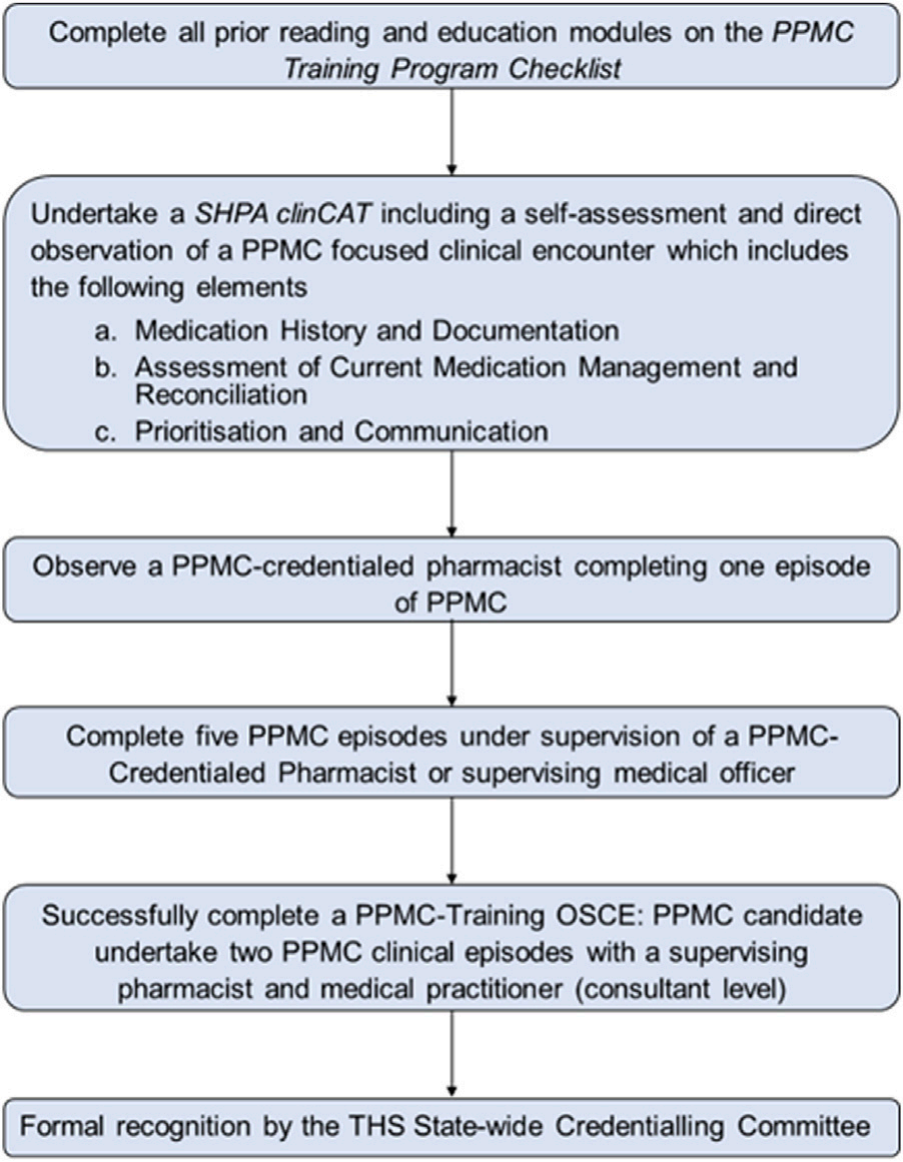
Summary of the credentialling pathway for partnered pharmacist medication charting credentialling candidates. Abbreviations: ClinCAT, clinical competency achievement tool; OSCE, objective structured clinical examination; PPMC, partnered pharmacist medication charting; SHPA, Society of Hospital Pharmacists of Australia; THS, Tasmanian Health Service.

PPMC operating model of care and credentialing process for Royal Hobart Hospital (RHH) were adapted from Victoria’s Alfred Hospital to fit the Tasmanian Health Service (THS) requirements (Tong et al., 2015). More information regarding the PPMC model is given elsewhere (Atey et al., 2023). Figure 1 outlines the credentialing pathway for eligible pharmacists, which included the final objective structured clinical examination (OSCE)-credentialing assessment.

### 2.2 Study setting

The PPMC project was implemented and evaluated in the RHH ED, a 490-bed teaching and referral public hospital located in southern Tasmania (Australia). The hospital is the largest hospital and the state’s major referral center that provides acute, sub-acute, mental health and aged care inpatient and outpatient services. It provides services to approximately a quarter-million people each year, with over 63,000 annual ED visits (Department of Health and Human Services, 2021).

### 2.3 Study design, population, and period

This study employed a controlled concurrent pragmatic evaluation design that compared three practicing models in a real-world setting simultaneously. The study included people aged 65 years or older, presenting to the RHH ED between 1 June 2020 and 17 May 2021.

### 2.4 Inclusion and exclusion criteria

Older people aged 65 years or above who presented to ED with subsequent admission to one of the three acute medical units (general medicine, emergency medicine or mental health unit), were taking at least one regular medication prior to hospitalization and received their first medication reconciliation (MedRec) on the ward within 48 h after transferring from ED were included. MedRec is defined as “*the formal process of obtaining and verifying a complete and accurate list of each patient’s current medicines and matching the medicines the patient should be prescribed to those they are actually prescribed in the hospital*” (Australian Commission on Safety and Quality in Health Care, 2011). Patients were ineligible if were not admitted to hospital after management in the ED, were admitted to units other than an eligible unit, had overnight ED presentations between 9 p.m. and 8 a.m. (i.e., outside of the PPMC Pharmacists working hours), did not receive MedRec within 48 h after transfer from ED, or had incomplete data, such as incomplete discharge summary.

### 2.5 Study arms

The study comprised three distinct arms: the PPMC arm, the early best-possible medication history (BPMH) arm and the usual care arm, representing a redesigned process, a modified process and a traditional standard of care, respectively. In the PPMC arm, a pharmacist documented a patient’s BPMH shortly after they arrived in the ED. The BPMH was collected through a structured patient interview and from secondary sources, such as caregivers, electronic health records, and community pharmacies. Following a clinical review, the pharmacist and a medical officer (a post-graduate year 2 resident or above) collaborated to develop a mutually agreed treatment plan. Based on this plan, the pharmacist charted the medications using purple ink, and each medication order was formally endorsed by the medical officer prior to its administration by the nursing staff.

The early BPMH included documentation of a BPMH by a pharmacist as early as feasible in the ED. This was then followed by the traditional medication charting approach, where a medical officer charted medications in the ED using black/blue ink. While the BPMH was available to the medical officer prior to charting, there was no clinical discussion between the pharmacist and the medical officer in this arm.

In the usual care arm, patients underwent the standard admission process, i.e., the traditional medication charting, where a medical officer wrote medication charts in the ED using black/blue ink. Notably, there was no pharmacist-collected BPMH or any collaborative discussions between the pharmacist and medical officer within the ED in this arm. Regardless of the study arms, ward clinical pharmacists offered standard clinical pharmacy services, including the conduct of MedRec, on the inpatient ward.

### 2.6 Data collection

A non-blinded independent researcher retrospectively collected the data from October 2020 to December 2021 by linking multiple datasets (i.e., ED presentation, MedRec, BPMH, admission and PPMC data) and accessing patients’ digital medical records. Data were also retrospectively collected from the Healthcare Software Clinical Suite (HCS), which is a working system that enables clinical pharmacists to record a BPMH and MedRec of the patients’ medicines. The researcher was not a member of the pharmacy team involved in PPMC, the admitting team or an RHH employee. Demographic, clinical and medication variables were collected through a predefined data collection form, which had received prior approval from an ethics committee. The extent of comorbidities was assessed using an age-adjusted Charlson comorbidity index (CCI), which takes into account 17 medical disorders and a patient’s age (Charlson et al., 1987). ED presentations were assessed using the Australasian Triage Scale (ATS), with 1 indicating the most critical presentation and 5 indicating the least critical presentation (Australasian College for Emergency Medicine, 2000).

### 2.7 Outcome measures

The 2019 American Geriatrics Society Beers Criteria (American Geriatrics Society Beers Criteria, 2019) were used to assess the use of PIMs at three different time points: a) medications taken before hospitalization (i.e., at baseline/presentation to ED), b) medications charted in the ED, and c) medications prescribed on hospital discharge. The primary outcome was the percentage of patients who were prescribed at least one PIM on ED departure. Secondary outcomes were the use of at least one PIM on hospital discharge and the number of PIMs in each group (i.e., the median number of PIMs per patient and the median number of PIMs per prescribed medication prescribed). The median number of PIMs per medication was obtained by dividing the total number of PIMs by the total number of medicines for each patient and then computing the median of this ratio for each study group. A relative risk reduction was also computed as the relative reduction in the risk of using at least one PIM in the PPMC group compared to the early BPMH group or the usual care group. For example,

$$\text{Relative risk reduction}=\frac{\text{Use}\;\text{of}\;\text{at}\;\text{least}\;\text{one}\;\text{PIM}\;\text{in}\;\text{the}\;\text{usual}\;\text{care}\;\text{group}\;\left(\%\right)\;‐\;\text{Use}\;\text{of}\;\text{at}\;\text{least}\;\text{one}\;\text{PIM}\;\text{in}\;\text{the}\;\text{PPMC}\;\text{group}\;\left(\%\right)}{\text{Use}\;\text{of}\;\text{at}\;\text{least}\;\text{one}\;\text{PIM}\;\text{in}\;\text{the}\;\text{usual}\;\text{care}\;\text{group}\;\left(\%\right)}\times 100$$

### 2.8 Sample size calculation

*A priori* sample size was calculated using a multigroup goodness-of-fit test with contingency tables in G*Power (V3.1.9.4, Westphalia, Germany). Considering 90% power, 5% significance and two degrees of freedom (i.e., a two-by-three contingency table depicting the binary primary outcome in each of the three study groups), 107 older people per group were required to detect a 20% relative reduction (i.e., moderate effect size) in the use of at least one PIM on ED departure with PPMC when compared to the early BPMH group or the usual care group. Samples of patients were selected randomly using an online random number generator (http://izmm.com/random.pl).

### 2.9 Data analysis

Categorical variables were summarized using frequencies and percentages. A normality test for continuous variables was determined using the Shapiro-Wilk test and graphical methods. Ordinal and non-normally distributed continuous data were presented using the median and interquartile range ([IQR]). Both between- and within-group analyses were conducted for comparisons of the PIM outcomes, i.e., use of at least one PIM, PIMs per patient and PIMs per medication. Categorical variables were compared between the groups using Pearson’s chi-square test or Fisher’s exact test, as appropriate. Between-group comparisons used the Kruskal–Wallis test with Dunn’s *post hoc* test. Within-group comparisons (on ED departure/hospital discharge vs*.* at baseline) used the Friedman rank-sum test with a pairwise Wilcoxon rank-sum *post hoc* test or Cochran’s Q test with Dunn’s *post hoc* test, as appropriate. A *p*-value of less than 0.05 was considered statistically significant in all analyses. The *p*-values were adjusted for multiple comparisons using the Benjamini–Hochberg method. The statistical analyses were conducted in R^®^ 4.1.12 (R Foundation for Statistical Computing, Vienna, Austria) (R Core Team, 2013).

Ethics approval from the University of Tasmania Human Research Ethics Committee (H0018682) and site authorization were obtained before commencing the study.

## 3 Results

### 3.1 Patients’ characteristics

**FIGURE 2:**
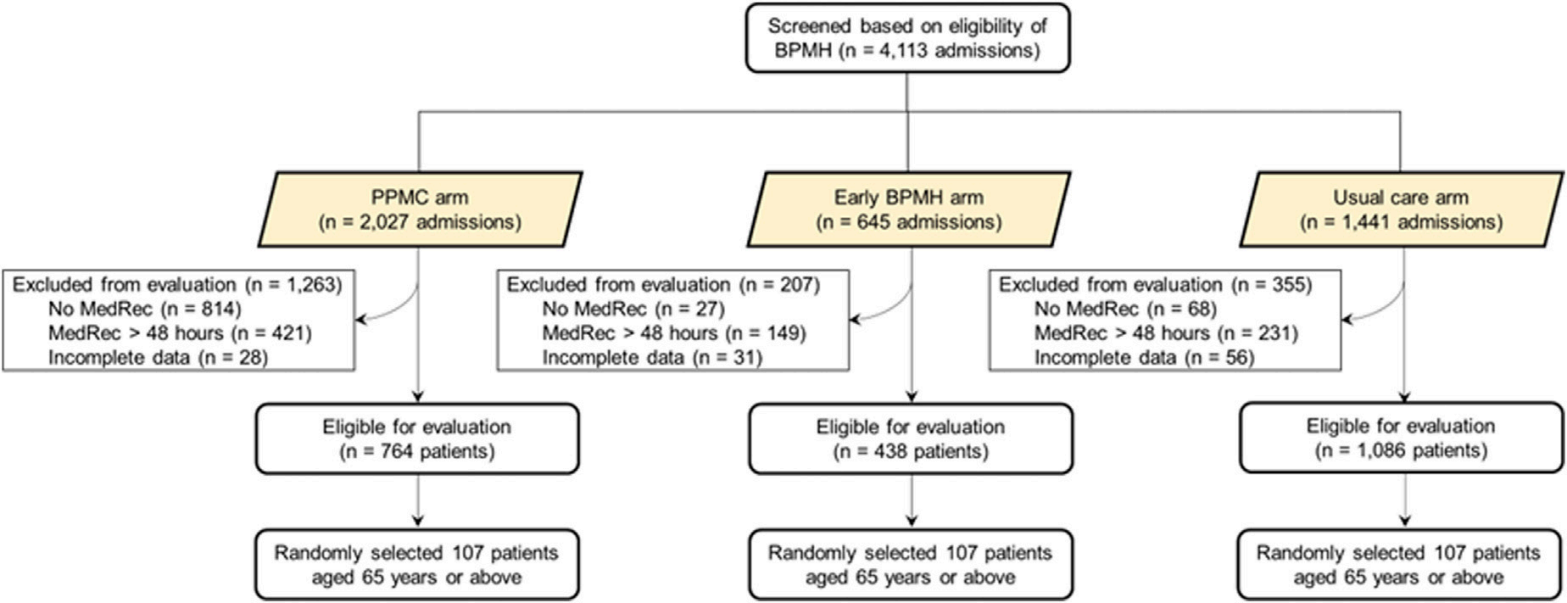
Flow chart depicting the selection of study patients. Abbreviations: AMU, acute medical unit; BPMH, best-possible medication history; ED, emergency department; MedRec, medication reconciliation; PPMC, partnered pharmacist medication charting. BPMH was limited to within 48 h post-admission (i.e., until the MedRec time) in the usual care arm.†Examples include incomplete/unavailable discharge summary or medication chart information. PPMC group included BPMH followed by a collaborative medication charting in ED. Early BPMH group included BPMH followed by a medical officer-led traditional medication charting approach in ED. Usual care group included BPMH on the inpatient ward after the traditional medication charting in ED.

During the study period, 62,662 patients presented to the RHH ED. Screening and selection of the study participants are presented in detail elsewhere (Atey et al., 2023). Three hundred twenty-one older people, 107 per group, were randomly selected and included in the analysis (Figure 2).

The groups differed with respect to age (*p* < 0.001), CCI (*p* = 0.039), number of initially charted medicines in the ED (*p* = 0.042) and admission units (*p* < 0.001). Median ages were 82.3, 80.1 and 75.4 years in the PPMC, early BPMH and usual care groups, respectively. Medicines charted in the ED were higher in the PPMC group than in the usual care group (*p* = 0.046) (Table 1).

**TABLE 1:** Baseline demographic and clinical characteristics of the study patients.

Characteristic, n (%) or median (interquartile range)	Study group			*p*-value	
	PPMC (N = 107)	Early BPMH (N = 107)	Usual care (N = 107)	Overall	Pairwise
Sex female	63 (59)	53 (50)	55 (51)	0.35^a^	
Age (years)	82.3 (75.2, 88.1)	80.1 (73.9, 84.6)	75.4 (70.7, 81.4)	**< 0.001** ^b^	0.06^‡^, **< 0.001** ^§^, **0.017** ^¶^
Comorbidity index	5 (5, 6)	5 (4.5, 6)	5 (4, 6)	**0.039** ^b^	0.24^‡^, **0.032** ^§^, 0.26^¶^
Triage scale	3 (2.5, 4)	3 (3, 3.5)	3 (2, 3)	0.49^b^	0.79^‡^, 0.57^§^, 0.77^¶^
Medicines					
Pre-admission (baseline)	10 (7, 13)	11 (6, 14)	9 (6, 12)	0.16^b^	0.96^‡^, 0.18^§^, 0.32^¶^
Initially charted in ED	11 (8, 14)	10 (8, 13)	9 (7, 12)	**0.042** ^b^	0.55^‡^, **0.046** ^§^, 0.10^¶^
Hospital discharge	9 (6, 13)	9 (6, 12)	8 (5, 12)	0.25^b^	0.54^‡^, 0.30^§^, 0.45^¶^
Acute admission units				**< 0.001** ^a^	
Cardiology	0 (0%)	9 (8.4%)	8 (7.5%)		
Emergency Medicine	7 (6.5%)	13 (12.1%)	15 (14%)		
General Medicine	100 (93.5%)	65 (60.7%)	44 (41.1%)		
Respiratory Medicine	0 (0%)	5 (4.7%)	16 (15%)		
Stroke	0 (0%)	5 (4.7%)	15 (14%)		
Others^c^	0 (0%)	10 (9.4%)	9 (8.4%)		
ED arrival to MedRec (hours)	24.8 (21.4, 32.0)	22.3 (12.6, 34.9)	27.4 (21.2, 44.1)	**0.002** ^b^	0.08^‡^, 0.11^§^, **0.001** ^¶^

^a^ Pearson’s chi-square test.

^b^ Kruskal–Wallis with Dunn’s *post hoc* tests: ‡PPMC, vs*.* early BPMH; §PPMC, vs*.* usual care; ¶early BPMH, vs*.* usual care.

^c^ Others: Endocrinology, Neurology, Renal Medicine, Rheumatology.

Bold Highlights values that have statistical significance.

### 3.2 Types of PIMs

Proton-pump inhibitors (PPI) (210 of 647, 32.5%), benzodiazepines (123 of 647, 19.0%) and antidepressants (117 of 647, 18.1%) were the most prevalent Beers PIM categories in all study groups over the three time points. PPIs, benzodiazepines and antidepressants accounted for 65.3% (130 of 199) of all PIM occurrences on ED departure: 41 of 52 (78.8%) in the PPMC group, 54 of 77 (70.1%) in the early BPMH group and 35 of 70 (50%) in the usual care group. Several PIM case vignettes are provided in Supplementary Appendix S1.

### 3.3 Use of PIMs between the study groups

**FIGURE 3:**
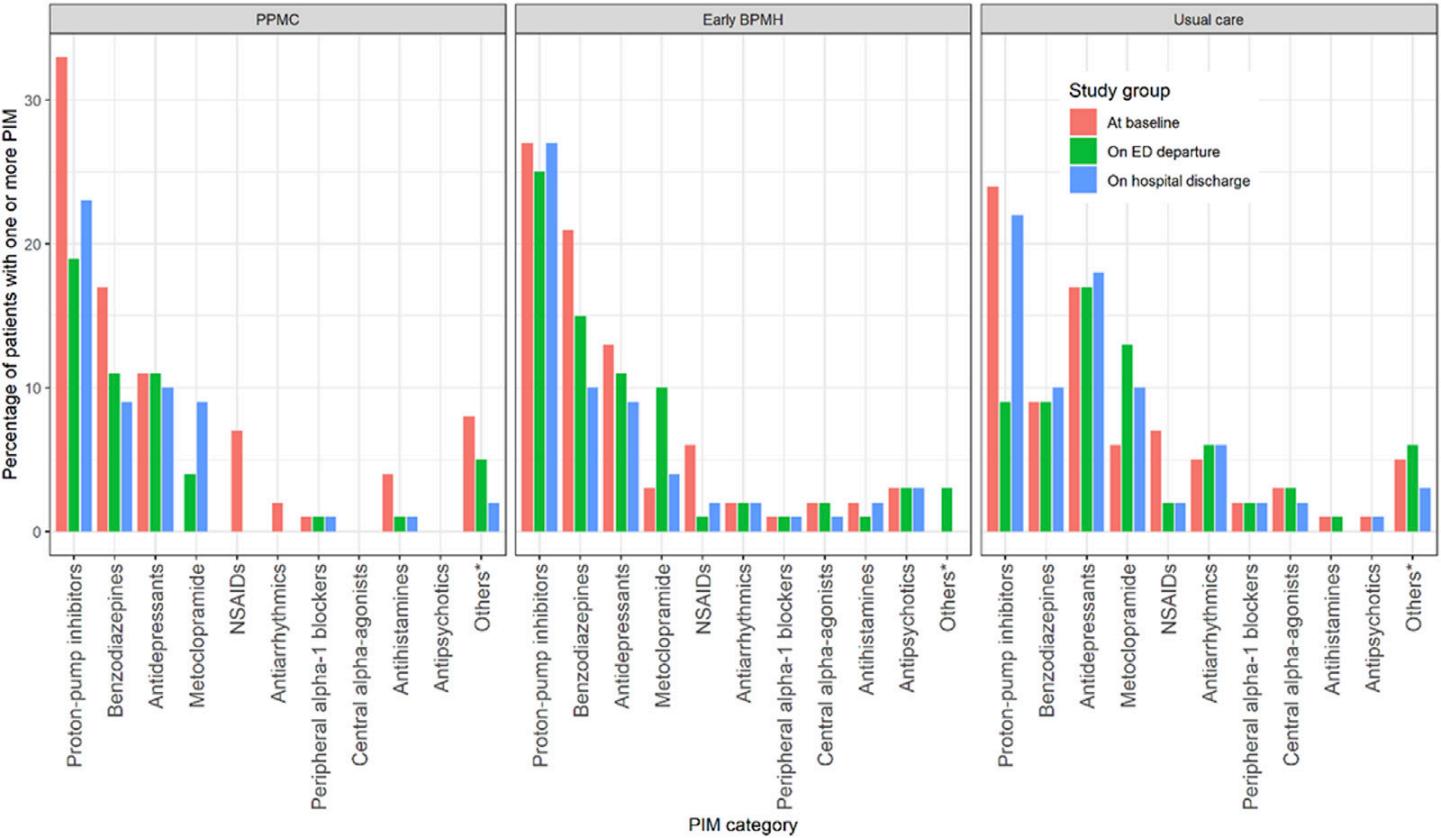
Use of at least one PIM by medication categories at each time point. Abbreviations: ED, emergency department; NSAIDs, nonsteroidal anti-inflammatory drugs; PIMs, potentially inappropriate medications *Others: Antiemetics, anti-infective, antispasmodics, estrogens, insulin, nonbenzodiazepine and sulfonylureas.

At baseline, the use of at least one PIM (*p* = 0.96), median number of PIMs per patient (*p* = 0.96) and median number of PIMs per medication (*p* = 0.84) were similar between the groups (Table 2). However, fewer patients in the PPMC group (41%) were prescribed at least one PIM, despite being prescribed more drugs, than those in the early BPMH group (48%) and the usual care group (51%) upon ED departure (*p* = 0.040). The risk of using at least one PIM on ED departure was reduced by 14.6% (95% confidence interval [CI]: 12.4%–17.8%) and 19.6% (95% CI: 16.8%–23.7%) with PPMC when compared to the early BPMH group and the usual care group, respectively. Use of at least one PIM from benzodiazepines, non-steroidal anti-inflammatory drugs, antiarrhythmics or antihistamines was largely reduced with PPMC upon ED departure and hospital discharge (Figure 3).

**TABLE 2:** Comparison of PIMs use between the groups and within each study group.

Outcomes		Study group			*Between-group comparison*	
		PPMC	Early BPMH	Usual care	Overall	Pairwise
**Patients prescribed ≥ 1 PIM**, n (%)						
Baseline		59 (55%)	55 (51%)	56 (52%)	0.92*	
ED departure		44 (41%)	51 (48%)	54 (51%)	0.040*	
Hospital discharge		46 (43%)	49 (46%)	53 (50%)	0.27*	
***Within-group comparison***	Overall	0.001**	0.35**	0.83**		
	Pairwise	**0.040** ^‡‡^, 0.113^§§^	0.88^‡‡^, >0.99^§§^	>0.99^‡‡§§^		
**PIMs per patient**, median (IQR)						
Baseline		1 (0, 1)	1 (0, 1)	1 (0, 1)	0.96^†^	0.89^‡§¶^
ED departure		0 (0, 1)	0 (0, 1)	1 (0, 1)	**0.036** ^†^	**0.046** ^‡§^, 0.88^¶^
Hospital discharge		0 (0, 1)	0 (0, 1)	0 (0, 1)	0.32^†^	0.54^‡¶^, 0.40^§^
***Within-group comparison***	Overall	**< 0.001** ^††^	0.09^††^	0.51^††^		
	Pairwise	**0.043** ^‡‡^, **0.046** ^§§^	0.77^‡‡^, 0.59^§§^	0.84^‡‡§§^		
**PIMs per medication**, median (IQR)						
Baseline		0.06 (0, 0.13)	0.05 (0, 0.12)	0.06 (0, 0.13)	0.84^†^	0.92^‡¶^, 0.97^§^
ED departure		0 (0, 0.07)	0 (0, 0.10)	0.05 (0, 0.11)	**0.029** ^†^	**0.042** ^‡§^ **,** 0.84^¶^
Hospital discharge		0 (0, 0.09)	0 (0, 0.11)	0 (0, 0.14)	0.28^†^	0.61^‡^, 0.35^§^, 0.43^¶^
***Within-group comparison***	Overall	**0.023** ^††^	0.22^††^	0.92^††^		
	Pairwise	**0.028** ^‡‡^, 0.09^§§^	0.70^‡‡§§^	0.88^‡‡§§^		

Abbreviations: ED, emergency department; IQR; interquartile range; PIM, potentially inappropriate medication.

The between-group comparison shows the comparison of PIM, outcomes between the three groups at a given time point (i.e., ‡PPMC, vs. early BPMH; §PPMC, vs*.* usual care; ¶early BPMH, vs*.* usual care) using the following tests.

•*Pearson’s chi-square test.

•†Kruskal–Wallis rank sum test for overall comparison and Dunn’s *post hoc* test for pairwise comparison.

The within-group comparison provides a comparison of PIM, outcomes for a specific study group on ED, departure/hospital discharge vs. baseline (i.e., ‡‡ED, departure vs*.* baseline; §§Hospital discharge vs*.* baseline) using the following tests.

• **Cochran’s Q test for overall comparison with Dunn’s *post hoc* test for pairwise comparison.

• ††Friedman rank sum test for overall comparison with multiple comparisons using pairwise Wilcoxon rank sum test.

Bold highlights values that have statistical significance.

On ED departure, the median number of PIMs per patient (*p* = 0.046) and the median number of PIMs per medication (*p* = 0.042) were both significantly lower for the PPMC group than for the comparison groups. However, the PIM outcomes did not differ significantly between the PPMC group and the comparison groups on hospital discharge. Likewise, there were no statistically significant changes in PIM outcomes on either ED departure or hospital discharge for the early BPMH group compared to the usual care group. Further PIM prevalence information is available in Supplementary Appendix S2.

### 3.4 Use of PIMs within each study group

Within-group analysis for the PPMC group showed statistically significant improvements in the use of at least one PIM on ED departure vs*.* at baseline (*p* = 0.040). By contrast, no significant changes were seen in the use of at least one PIM on ED departure vs*.* at baseline within the early BPMH group (*p* = 0.88) or the usual care group (*p* > 0.99). The percentage of patients who were prescribed at least one PIM did not change significantly within each of the study groups on hospital discharge when compared to the baseline.

Unlike the median number of PIM per patient, no significant changes were seen in the median number of PIM per prescribed medication on hospital discharge vs*.* at baseline within the PPMC group. Neither the median number of PIM per patient nor the median number of PIM per prescribed medication changed significantly within the comparison group on hospital discharge vs*.* at baseline (Table 2).

## 4 Discussion

Compared to early BPMH alone or usual care, the use of PIMs was significantly reduced with PPMC in the ED, where the model of care was implemented, but not upon hospital discharge. The within-group analysis indicated statistically significant decreases in PIMs use on ED departure compared to presentation within the PPMC group, and the impact lasted until hospital discharge for the median number of PIMs only. None of the PIM outcomes on ED departure or hospital discharge differed significantly from baseline within the comparison groups. The ED findings were consistent with literature that reported an association between ED-based pharmacist interventions and improved appropriateness of prescribed medications in the ED (Airaksinen et al., 2021; Atey et al., 2022).

The clinical discussion held between PPMC-credentialled pharmacists and medical officers in the ED was the main differentiating factor between the PPMC arm and the comparison arms. Patient-specific clinical information and medication issues were the primary topics of conversation. Presumably, the observed reductions in the use of PIMs in the ED with PPMC were primarily driven by the collaborative face-to-face clinical discussion.

There were no significant differences between the PPMC group and the comparison groups for any of the PIM outcomes on hospital discharge, as opposed to the findings on ED departure. Charting issues (e.g., drug omission errors) and clinical issues (e.g., inappropriate drugs) were more likely to be identified in the ED through the PPMC model of care in the PPMC group and on the ward through the MedRec model in the comparison groups (Tesfaye et al., 2019; Atey et al., 2023). While MedRec is likely to continue ensuring the safety of medications on the inpatient ward, MedRec alone may be limited in its capacity to achieve statistically significant reductions in the use of PIM on hospital discharge vs*.* at baseline within the comparison groups. This could be because, unlike in the PPMC model, pharmacists’ face-to-face discussions with medical officers were not a routine component of the MedRec model. Participation of pharmacists in direct therapeutic dialogues with prescribers may improve the implementation of pharmacists’ recommendations (Gillespie et al., 2009).

The use of some PIMs, mainly PPI, was resumed on the inpatient ward after not being prescribed in the ED. Although the benefit vs*.* risk of resuming a PIM requires a case-by-case clinical decision, which is beyond the scope of this study and a potential area for future research, this finding further collaborated the value of implementing shared decision-making on the ward to sustain treatment optimization. It is also possible that a patient may be taking a PPI while on a corticosteroid in the ED, which would not qualify the PPI to be a PIM according to the Beers Criteria. However, the PPI could be considered a PIM if it were continued without the corticosteroid on the ward and afterwards.

### 4.1 Clinical implication

The presence of PPMC pharmacists and their collaboration with medical officers in ED significantly reduced older ED patients’ exposure to PIMs. However, our study found no statistically significant difference between the three study groups in PIM use at hospital discharge, supporting the need for a MedRec on the inpatient ward, but with a closer interprofessional collaboration approach, as in PPMC. Ensuring medication safety in a hospital, thus, requires a continuum of close collaboration among health professionals that takes into account the dynamic hospital medication use process or evolving patient clinical status (Hughes and Blegen, 2008; Hua et al., 2022). In a complex healthcare system, potential medication errors are prevented from causing harm by placing a successive, series of layers of medication safety safeguards across the transition of care, similar to a Swiss cheese model (Reason, 2000).

### 4.2 Strengths and limitations

To avoid organizational bias, this evaluation study was conducted by an external, independent research group. Another strength of the study was a simultaneous comparison of the three arms, which provided a method to concurrently assess the impact of different ED-based care models. We assessed the use of PIMs at three-time points using a controlled concurrent design. Adding both baseline and control groups for comparison may help overcome the confounding issues in non-randomized studies (Harris et al., 2006).

PIM use was assessed using Beers criteria, which is an explicit, criterion-based assessment tool that requires minimal clinical judgment and is inexpensive. By contrast, implicit criteria, such as medication appropriateness index (Hanlon et al., 1992), are subjective metrics that are time-consuming and complex (Lopez-Rodriguez et al., 2020). It is important to acknowledge that at the time of manuscript writing, the updated version of the Beers Criteria 2023 had not been released, making it challenging to consider the updated criteria in our analysis. Future studies should consider the 2023 Beers Criteria when evaluating the impact of PPMC on the use of PIM.

Without randomized allocation of the study participants, it is still possible that all variables might not be adequately controlled with a possible risk of residual confounding, although the use of PIMs was similar at baseline across the groups. Patients in the three study groups had different baseline demographic and clinical characteristics (Table 1). Compared to patients in the usual care group, those in the PPMC group were older, had more medically complex conditions and had a higher number of initially charted medicines. This difference may suggest that the hospital may have purposely enrolled these patients in the PPMC arm with the expectation that they would gain the utmost benefit from their participation.

Blinding of data collection was not possible as patients could easily be identified during the retrospective data collection. As a retrospective study, there may be a possibility that the data may not have been recorded completely and consistently (Cole and Trinh, 2017).

## 5 Conclusion

Compared to early BPMH alone or usual care, the co-charting model significantly reduced the use of PIMs on ED departure among older patients who presented to ED with at least one regular pre-admission medication. However, outcomes at hospital discharge were not statistically different. No significant changes were seen in any PIM outcomes on ED departure or hospital discharge vs*.* at baseline within the comparison groups. The findings may support the continuance of the model in the ED setting to ensure appropriate medication use in the hospital, with a similar collaborative approach needed on the wards.

## Data Availability

The data analyzed in this study were obtained from the Tasmanian Government Department of Health and collected from patients’ records. Human Research Ethics Committee and site authorization approvals are required to access the data. The datasets presented in this article are not readily available because only individuals named in the ethics application are authorized to access the data. Requests to access the datasets should be directed to Dr Mohammed Salahudeen at Mohammed.Salahudeen@utas.edu.au.
