# Supplementary material for: The impact of partnered pharmacist medication charting in the emergency department on the use of potentially inappropriate medications in older people
Source: Front Pharmacol. 2023 Nov 7;14:1273655. doi: 10.3389/fphar.2023.1273655 (PMC10664652; doi:10.3389/fphar.2023.1273655)
Supplement: Supplementary file 1 [file Table1.docx]

**Supplementary Appendix**

The impact of partnered pharmacist medication charting in the emergency department on the use of potentially inappropriate medications in older people

**Supplementary Appendix S1. Potentially inappropriate medication case vignettes.**

| **Cases** | **Medication** | **Case description** | **PIM definition using the Beers criteria (American Geriatrics Society Beers Criteria, 2019)** |
| --- | --- | --- | --- |
| Case 1 | Esomeprazole | A patient, in their early 70s, was on chronic esomeprazole for gastro-esophageal reflux disease for more than a year. The patient was not a high-risk patient, i.e., not on any oral corticosteroid or chronic NSAIDs use, had no history of erosive esophagitis, Barrett’s esophagitis, pathological hypersecretory condition, or no history of failure of drug discontinuation trial or H_2_-receptor antagonists. | Because of the risk of bone loss and fractures and *Clostridium difficile* infection, scheduled use of esomeprazole for more than eight weeks should be best avoided for the patient unless they are high-risk patients. |
| Case 2 | Digoxin | A patient, in their mid-80s, presented to ED after being found on the floor at their home, presumed due to urosepsis. The patient was taking digoxin 0.185 mg each morning prior to hospitalisation for their paroxysmal atrial fibrillation. The same dosage was continued in ED. | Dosages more than 0.125 mg are recommended to be avoided for the patient because higher dosages are not associated with additional benefits and may increase the risk of toxicity. |
| Case 3 | Amitriptyline | A patient, in their late 80s, presented to ED with chest heaviness due to rapid atrial fibrillation, on a background of right lacunar cerebrovascular accident, hypertension and anxiety. The patient had been prescribed amitriptyline 10mg each evening for sleep. | Amitriptyline should be best avoided for the patient because of its highly sedating and anticholinergic effect (e.g., it could worsen tachycardia), and causation of orthostatic hypotension. |

Abbreviations: ED, emergency department; NSAIDs, non-steroidal anti-inflammatory drugs; PIM, potentially inappropriate medication
