# Supplementary material for: The impact of partnered pharmacist medication charting in the emergency department on the use of potentially inappropriate medications in older people
Source: Front Pharmacol. 2023 Nov 7;14:1273655. doi: 10.3389/fphar.2023.1273655 (PMC10664652; doi:10.3389/fphar.2023.1273655)
Supplement: Supplementary file 2 [file Table2.docx]

**Supplementary Appendix**

The impact of partnered pharmacist medication charting in the emergency department on the use of potentially inappropriate medications in older people

**Supplementary Appendix S2. Use of potentially inappropriate medications between the study groups.**

| **Outcomes** | **Study group** | | |
| --- | --- | --- | --- |
|  | **PPMC**  (n = 107) | **Early BPMH**  (n = 107) | **Usual care**  (n = 107) |
| **Medications**, total |  |  |  |
| Baseline | 1148 | 1147 | 1003 |
| ED departure | 1172 | 1157 | 1025 |
| Hospital discharge | 1033 | 991 | 932 |
| **PIM**, total |  |  |  |
| Baseline | 83 | 83 | 86 |
| ED departure | 52 | 77 | 73 |
| Hospital discharge | 55 | 64 | 79 |
| **PIM use, n (%)** |  |  |  |
| **Baseline** |  |  |  |
| **Use of at least one PIM** | 59 (55%) | 55 (51%) | 56 (52%) |
| 0 PIM | 48 (45%) | 52 (49%) | 51 (48%) |
| 1 PIM | 41 (38%) | 35 (33%) | 36 (34%) |
| 2 PIM | 13 (12%) | 15 (14%) | 14 (13%) |
| 3 PIM | 4 (3.7%) | 3 (2.8%) | 2 (1.9%) |
| ≥ 4 PIM | 1 (0.9%) | 2 (1.9%) | 4 (3.7%) |
| **ED departure** |  |  |  |
| **Use of at least one PIM** | 44 (41%) | 51 (48%) | 54 (50%) |
| 0 PIM | 63 (59%) | 56 (52%) | 53 (50%) |
| 1 PIM | 37 (35%) | 28 (26%) | 40 (37%) |
| 2 PIM | 6 (5.6%) | 20 (19%) | 10 (9.3%) |
| 3 PIM | 1 (0.9%) | 3 (2.8%) | 3 (2.8%) |
| ≥ 4 PIM | 0 (0%) | 0 (0%) | 1 (0.9%) |
| **Hospital discharge** |  |  |  |
| **Use of at least one PIM** | 46 (43%) | 49 (46%) | 53 (50%) |
| 0 PIM | 61 (57%) | 58 (54%) | 54 (50%) |
| 1 PIM | 38 (36%) | 37 (35%) | 34 (32%) |
| 2 PIM | 7 (6.5%) | 9 (8.4%) | 15 (14%) |
| 3 PIM | 1 (0.9%) | 3 (2.8%) | 1 (0.9%) |
| ≥ 4 PIM | 0 (0%) | 0 (0%) | 3 (2.8%) |

Abbreviations: ADRs, adverse drug reactions; BPMH, best-possible medication history; ED, emergency department; PIM, potentially inappropriate medication; PPMC, p
